# The impact of US policy on contraceptive access: a policy analysis

**DOI:** 10.1186/s12978-021-01289-3

**Published:** 2021-11-22

**Authors:** Laura E. T. Swan

**Affiliations:** Department of Population Health Sciences, University of Wisconsin-Madison, Madison, WI USA

**Keywords:** Family planning, Reproductive health, Healthcare access, Grey literature, Contraceptive care

## Abstract

**Background:**

Contraceptive access is influenced by policy decisions, which can expand and constrict the contraceptive options available. This study explored the impact of recent US federal policy on contraceptive access.

**Methods:**

Federal policy changes impacting contraceptive access over the past decade were identified in grey literature. These policy changes were organized into a timeline and analyzed according to Levesque et al.'s (2013) five dimensions of healthcare access (approachability, acceptability, availability/accommodation, affordability, and appropriateness), noting the most salient healthcare dimension impacted by the policy change and analyzing whether, according to this framework, the policy created a theoretical increase or decrease in contraceptive access.

**Results:**

Of those policy changes coded as increasing (*n* = 42) and decreasing (*n* = 28) contraceptive access, most were related to the affordability (increasing *n* = 13; decreasing *n* = 12), physical availability (increasing *n* = 10; decreasing *n* = 7), and appropriateness (increasing *n* = 12; decreasing *n* = 4) of contraceptive care. Policy changes largely followed partisan divides, with contraceptive access increasing in years with a Democratic president and decreasing when a Republican president was in office. Many policy changes were related to the Affordable Care Act (ACA) and Title X of the Public Health Services Act. The implementation of the ACA and subsequent updates to it have increased the affordability of contraception, whereas changes to Title X have decreased the availability and appropriateness of contraceptive care.

**Conclusions:**

This study highlights recent policy changes impacting contraceptive access, organizing them according to the five dimensions of healthcare access. It outlines specific policy barriers to contraceptive access and provides suggestions for policy and practice action that will improve contraceptive access and reproductive autonomy. Opportunities to ensure contraceptive access for all Americans include promoting comprehensive sex education, extending the Community Health Center Fund, increasing contraceptive care options for people with employers who are exempted from the ACA contraceptive mandate, addressing discrimination and building trust in contraceptive care, and amplifying outreach efforts to combat misinformation and confusion created by continuous changes to key family planning policies. Continued research on the role of policy in determining reproductive autonomy is warranted, and practice and policy action is needed to improve contraceptive access.

## Plain language summary

Legislation influences if, when, and how people access contraception. Healthcare access can be conceptualized across five dimensions: approachability, acceptability, availability/accommodation, affordability, and appropriateness. Applying this healthcare access framework can help understand barriers to and facilitators of contraceptive access. In this study, I conducted a systematic Google search to identify recent federal policy changes related to contraceptive access. I organized these policy changes into a timeline and analyzed them according to the healthcare access framework mentioned above. Through this analysis, I have highlighted specific policy barriers to contraceptive access and provided suggestions for policy and practice action that can improve contraceptive access and reproductive autonomy.

Findings indicated that Levesque et al. (2013) dimensions of healthcare access are relevant to contraceptive care. Many recent US policy changes related to contraceptive access are described in this study, and the analysis showed that these policy changes have fluctuated with election cycles and according to partisan divides. Two key US policies related to contraceptive access, the Affordable Care Act and Title X of the Public Health Services Act, have sustained many changes across the past decade. These policies have increased contraceptive access in the United States by dedicating funding to family planning availability and affordability and increasing insurance coverage for contraceptive care. However, recent changes to these policies threaten contraceptive access and reproductive autonomy. There is a need for continued research regarding the ways that policy influences reproductive autonomy and a need for practice and policy action to improve contraceptive access.

In the United States, nearly half of pregnancies are unintended [1–3], and a majority of reproductive-aged women are at risk of unintended pregnancy [4]. Although correct and consistent use of contraception reduces the risk of unintended pregnancy to 5% [5], many Americans face challenges to accessing contraception, citing barriers such as affordability [6–8], shame or embarrassment [9, 10], and difficulty physically reaching services [7, 11]. Furthermore, barriers to care disproportionately impact populations who are already marginalized based on factors such as age, income, race/ethnicity, rurality, education level, or exposure to violence [2, 11–13].

In addition, policy decisions expand and constrict the contraceptive options available. For example, in 1873, the Comstock Act limited contraceptive access by marking contraceptives as obscene and criminalizing their distribution [14] until nearly a century later in the 1965 Supreme Court decision in *Griswold v. Connecticut* [14, 15]. Meanwhile, eugenics practices flourished and were affirmed in the 1927 Supreme Court decision in *Buck v. Bell* which upheld the legality of forced sterilization [14, 16]. In 1942, in *Skinner v. Oklahoma,* the Supreme Court ruled against compulsory sterilization of convicted criminals but did not address the forced sterilization of other populations (e.g., based on income, minority status, or mental illness) [14, 15, 17]. These practices continued for many years, and reports of the forced sterilization of imprisoned populations and coercion involving incentives to promote permanent or long-acting contraception continue to emerge [16, 18]. The eugenics movement also helped propel the development and distribution of the contraceptive pill, which was tested on Puerto Rican women in the 1950s and has been used as a method of population control, targeting groups whose reproduction was considered “undesirable” [19, 20].

Other policies have increased contraceptive access by creating funding streams that help make family planning services more available and affordable. For example, Title X of the Public Health Services Act of 1970 (Title X) established federal funding for family planning services [14, 21]. This policy has increased contraceptive access by providing funds to community-based health facilities across the country, allowing for more affordable contraceptive care to be provided in convenient locations. Similarly, when Medicaid was expanded in 1972 to fund family planning services and supplies [22], contraceptive access increased by improving the availability and affordability of contraception.

In recent years, reproductive politics in the US have been at the forefront of polarized political debate, with many Democrats and Republicans differing significantly in their view on topics including abortion and contraception [23, 24]. Typically, Democrats are more aligned with progressive policies that promote family planning access, whereas Republicans often promote legislation that aligns with social and fiscal conservatism [25–28]. Debates over health policy have occurred amongst shifts in political power which influence the proposal and passage of family planning legislation [23, 29]. In roughly the past decade, the US presidential office has passed from Republican George W. Bush to Democrat Barack Obama in 2009, then to Republican Donald Trump in 2017, and most recently, to Democrat Joe Biden in 2021 [30]. In addition, the majority party in the US Senate and House of Representatives shifts over time, most recently passing from a Democratic Senate majority from 2009 to 2015 to a Republican Senate majority from 2015 to 2021 and a from a Democratic House majority from 2009 to 2011 to a Republican House majority from 2011 to 2019 and finally to a Democratic House majority from 2019 to 2021 [31, 32]. These power shifts greatly influence policy decisions [29] which can create or prevent contraceptive access.

## Current study

Earlier works [14] have reviewed and analyzed the history and impact of US reproductive politics prior to important recent changes in family planning legislation. To provide a comprehensive and overarching understanding of the state of contraceptive care in the United States and to identify needs for policy and practice action, there is a need for research that consolidates and analyzes the role of more recent policy changes in determining reproductive healthcare access and contraceptive access, specifically. Levesque et al.'s (2013) healthcare access framework can be applied to concretely examine the individual and system-level factors that determine contraceptive access [33]. This framework conceptualizes access to healthcare as determined by five dimensions (approachability, acceptability, availability and accommodation, affordability, and appropriateness) that interact to generate healthcare access. See Table 1 for a description of this healthcare access framework and application of its dimensions to contraceptive care.

**Table 1:** Description of Healthcare Access Framework and Application to Contraceptive Care

Dimension^a^	Description^a^	Application to Contraceptive Care
Approachability	The ability to perceive the need for care. Related to community members’ health knowledge and health literacy, the transparency of available health services, and providers’ outreach endeavors.	• Sex education • Accuracy of contraceptive knowledge • Information about available family planning services and how and where to access this care
Acceptability	The ability to seek care. Related to cultural and social factors that determine how people think and feel about healthcare services.	• Beliefs, social norms, stigma, and fear of judgment surrounding sex, contraceptive use, and pregnancy • Comfort with family planning conversations • Decision-making priorities •Trust in family planning providers and the confidentiality of care
Availability and Accommodation	The ability to physically reach care in a timely manner. Related to the geographic location of services, the hours of operation and availability of appointments, facility accessibility, and availability of transportation needed to reach care.	• Family planning clinics’ physical location and health center density • Shortages in family planning providers • Limited clinic hours of operation • Same-day, on-site availability of contraceptive services
Affordability	The ability to pay for care. Related to the price of health services and community members’ income and other assets such as time, health insurance, and social capital.	• Cost and insurance coverage of contraceptive care • Patients’ income and access to health insurance • Local and federal family planning funding
Appropriateness	The ability to engage with care. Related to the fit between patient needs and the care offered, how adequately providers are trained to meet patient needs, and the interpersonal quality of the care provided.	• Providers’ ability to meet the contraceptive needs and priorities of their community • Provider’s family planning knowledge and training • Provider preparedness to provide comprehensive and unbiased contraceptive care • On-site availability of multiple contraceptive options •Providers’ decision-making model

^a^Adapted from Levesque et al. [33]

Applying Levesque et al. (2013) healthcare access framework [33] to explore the role of US policy in determining contraceptive access, the following research question guided this study: How have federal US policy changes from 2009 to 2019 impacted contraceptive access? To answer this question, I identified relevant policy changes and organized them into a policy timeline, analyzing these policy changes according to Levesque et al.’s (2013) five dimensions of healthcare access [33].

## Methods

In May 2020, I used Google to systematically search grey literature (i.e., government, academic, business, and industry works not controlled by commercial publishers) [34] for websites, news articles, and reports that discussed recent (passed from 2009 to 2019) US federal policy influences on contraceptive access. Utilizing grey literature sources allowed me to capture policy-related landmarks that impact contraceptive access regardless of whether the policies themselves directly include language about contraception and healthcare access. This also allowed for the identification of incremental and recent policy changes not yet represented in peer-reviewed literature. I used keywords related to contraceptive access (i.e., “family planning” OR contraception OR “birth control”) and healthcare access, as conceptualized by Levesque et al. (2013; e.g., transparency, transportation, insurance) [33].

I reviewed a total of 150 search results, including the first 30 results from a search with no year specified and the first 10 results from searches of sources published each year from 2009 to 2020. Of these, 65 sources were excluded for being duplicate sources, irrelevant to the topic at hand, not qualifying as grey literature, or discussing policies that were state-level, international, or passed before the target date of 2009. This left 85 grey literature sources (full list available upon request). Using these sources, I recorded any US federal policy change passed from 2009 to 2019 that was discussed as relevant to contraceptive access, and I noted a brief description of each policy. Then, I organized these policy changes into a timeline and coded them according to Levesque et al.'s (2013) dimensions of healthcare access [33], noting the most salient healthcare dimension impacted by the policy change and analyzing whether, according to this framework, the policy created a theoretical increase or decrease in contraceptive access. Through this process, I identified, analyzed, and organized specific policy barriers to contraceptive access, allowing me to present suggestions for policy and practice action that should improve contraceptive access and reproductive autonomy.

## Findings

Table 2 shows the policy timeline created using grey literature. This timeline provides the date and brief description of 77 US federal policy changes occurring between the years of 2009 and 2019, each described in grey literature as impacting contraceptive access. These policy changes were coded according to the most salient healthcare access category and according to whether they increased or decreased overall contraceptive access. Several (*n* = 7) political changes and appointments (e.g., Donald Trump being inaugurated as US president in January 2017) are also included in the policy timeline as relevant policy landmarks but are not coded according to access category because they would likely only impact contraceptive access indirectly via subsequent policy changes. I coded 42 policy changes as increasing contraceptive access and 28 as decreasing contraceptive access. Policy changes thought to increase contraceptive access were most commonly coded as relevant to affordability (*n* = 13), followed by appropriateness (*n* = 12), availability and accommodation (*n* = 10), approachability (*n* = 5), and acceptability (*n* = 2). Policy changes thought to decrease contraceptive access were most commonly coded as affordability (*n* = 12), followed by availability and accommodation (*n* = 7), appropriateness (*n* = 4), approachability (*n* = 3), and acceptability (*n* = 2).

**Table 2:** Policy Timeline: Changes in Federal US Policy (2009–2019) Impacting Contraceptive Access

Date	Policy Change	Theoretical Impact ‡
Jan 2009	Barack Obama inaugurated as US president	-
Feb 2009	Health Information Technology for Economic and Clinical Health (HITECH) enacted as part of the American Recovery and Reinvestment Act of 2009, increasing Health Insurance Portability and Accountability Act (HIPAA) privacy/security	↑ Approachability
Apr 2009	The Food and Drug Administration (FDA) lowers over-the-counter emergency contraceptive age to 17 years old	↑ Availability/ Accommodation
Jun 2009	Title V abstinence-only-until-marriage program expires	↑ Approachability
Dec 2009	Mikulski’s Women’s Health Amendment to the Patient Protection and Affordable Care Act (ACA) passes, adding women’s preventive care as mandated services	↑ Affordability
Dec 2009	Consolidated Appropriations Act of 2010 creates Teen Pregnancy Prevention Program	↑ Appropriateness
Mar 2010	ACA signed into law	↑ Affordability
Mar 2010	ACA amended through the Health Care and Education Reconciliation Act (HCERA) to include student loan reform, close the Medicare Part D donut hole, increase Medicaid payment rates, and expand Medicaid	↑ Affordability
Jun 2010	US Medical Eligibility Criteria for Contraceptive Use published to provide recommendations on safe use of contraceptive methods	↑ Appropriateness
Dec 2010	Healthy People 2020 establishes federal prevention agenda	↑ Appropriateness
Dec 2010	Consolidated Appropriations Act of 2011 ends Community-Based Abstinence Education grant program and eliminates abstinence-only portion of the Adolescent Family Life Act	↑ Approachability
Jan 2011	National Defense Authorization Act of 2011 creates TRICARE Young Adult, extending military member dependent coverage	↑ Affordability
Apr 2011	“Dear Colleague letter” sent to colleges and universities regarding new Title IX guidance on student harassment	↑ Acceptability
Aug 2011	ACA interim final rules announced in which the US Department of Health and Human Services (HHS) adopts IOM women’s preventive care guidelines, adding contraceptive coverage and preventive services to those covered by the ACA	↑ Affordability
Dec 2011	HHS overrules FDA decision to make emergency contraception available over the counter regardless of age	↓ Availability/ Accommodation
Dec 2011	Consolidated Appropriations Act of FY 2012 revives federal funding for abstinence-only programs by establishing the Competitive Abstinence Education (CAE) grant program	↓ Approachability
Jan 2012	Final rule church exemption from ACA contraceptive requirement announced	↓ Affordability
Feb 2012	Final rule ACA church exemption revised, extending it to other religious employers	↓ Affordability
Jun 2012	*National Federation of Independent Business v. Sebelius* Supreme Court of the United States (SCOTUS) ruling upholding most ACA provisions but making Medicaid expansion optional for states	↓ Affordability
Aug 2012	ACA contraceptive mandate implementation begins	↑ Affordability
Jan 2013	Final Omnibus Rule fills gaps in existing HIPAA and HITECH regulations, increasing privacy/security	↑ Approachability
Jun 2013	US Selected Practice Recommendations published to provide recommendations on how to use contraceptive methods safely and effectively	↑ Appropriateness
Jun 2013	Safe Harbor rule announced, updating religious exemption to ACA contraceptive mandate	↓ Affordability
Mar 2013	Campus Sexual Violence Elimination (Campus SaVE) Act integrated into the Violence Against Women Reauthorization Act of 2013, increasing requirements for colleges for sexual assault response and increasing survivors’ rights	↑ Acceptability
Apr 2013	Federal judge rules in Tummino v. Hamburg, removing age and point-of-sale restrictions on levonorgestrel-based emergency contraception, though 3-year market exclusivity was granted for Plan B®	↑ Availability/ Accommodation
Jan 2014	Medicaid expansion goes into effect	↑ Affordability
Apr 2014	Providing Quality Family Planning Services recommendations published, defining core services offered by family planning clinics	↑ Appropriateness
Jun 2014	*Burwell v. Hobby Lobby Stores, Inc.* SCOTUS ruling that for-profit companies with a religious objection to birth control are exempt from ACA contraceptive mandate	↓ Affordability
Aug 2014	Veterans Access, Choice, and Accountability Act of 2014 allows Veterans with specific burdens to receive healthcare with Choice contracted non-VA providers	↑ Availability/ Accommodation
Jan 2015	Navy increases availability of long-acting reversible contraception (LARC) during basic training and added walk-in contraceptive clinics	↑ Availability/ Accommodation
Apr 2015	Medicare and Children's Health Insurance Program Reauthorization Act (MACRA) of 2015 passes, extending Community Health Center Fund for two years	↑ Availability/ Accommodation
May 2015	HHS clarifies contraceptive methods covered under the ACA as preventive services	↑ Affordability
Oct 2015	Indian Health Service increases over the counter emergency contraception accessibility to Indigenous people	↑ Appropriateness
Dec 2015	Consolidated Appropriations Act of 2016 renames “Competitive Abstinence Education” program “Sexual Risk Avoidance Education” program	↓ Approachability
Jan 2016	Marines restrict availability of LARCs at basic training and begin promoting injectable contraceptives over other methods	↓ Availability/ Accommodation
Mar 2016	Providing Quality Family Planning Services recommendations revised	↑ Appropriateness
Apr 2016	Plan B® market exclusivity expires, allowing all generic emergency contraceptives to be available over the counter without age or point-of-sale restrictions	↑ Availability/ Accommodation
May 2016	*Zubik v Burwell* SCOTUS ruling sends 7 cases brought by religious nonprofits back to Courts of Appeal	↑ Affordability
Jul 2016	US Medical Eligibility Criteria for Contraceptive Use updated	↑ Appropriateness
Jul 2016	US Selected Practice Recommendations updated	↑ Appropriateness
Dec 2016	Title X eligibility requirements amended, prohibiting exclusion from subawards for reasons other than ability to provide services	↑ Availability/ Accommodation
Jan 2017	Donald Trump inaugurated as US president	-
Jan 2017	Anti-birth-control Katy Talento appointed to the White House Domestic Policy Council	-
Apr 2017	Neil Gorsuch confirmed as SCOTUS justice	-
Apr 2017	Joint Resolution of Disapproval nullifies Dec 2016 Title X eligibility amendment	↓ Availability/ Accommodation
May 2017	Antiabortion activist Teresa Manning appointed to lead Title X programs	-
Jun 2017	Abstinence-only advocate Valerie Huber appointed HHS chief of staff to the assistant secretary for health	-
Jul 2017	Funding for Teen Pregnancy Prevention Program cut two years before grants were scheduled to end	↓ Appropriateness
Sept 2017	Title IX guidance on student harassment (established in 2011 “Dear Colleague letter”) eliminated	↓ Acceptability
Sept 2017	Title V abstinence-only program expired briefly	↑ Approachability
Oct 2017	Interim rules released, extending religious exemption from ACA contraceptive mandate	↓ Affordability
Dec 2017	Interim rules (extending religious exemption from ACA contraceptive mandate) challenged and blocked from implementation pending litigation	↑ Affordability
Feb 2018	Bipartisan Budget Act of 2018 extends Community Health Center Fund for two more years ^†^	↑ Availability/ Accommodation
Feb 2018	Bipartisan Budget Act of 2018 rebrands and renews Title V abstinence-only program under new name “sexual risk avoidance education" program	↓ Approachability
Feb 2018	Call for Title X funding applications radically shifts Title X program, emphasizing natural family planning over comprehensive and evidence-based care	↓ Appropriateness
Feb 2018	Strategic Plan for 2018–2022, which states that life begins at conception, finalized as a guide for federal policy	↓ Acceptability
Apr 2018	Funding Opportunity Announcements shift Teenage Pregnancy Prevention Program to promote abstinence-only sex education	↓ Appropriateness
Apr 2018	District Court rules in Planned Parenthood of Greater Washington and North Idaho et al. v. HHS in favor of Planned Parenthood regarding Teenage Pregnancy Prevention grant termination	↑ Appropriateness
May 2018	Domestic gag rule (AKA “Protect Life Rule”) announced, proposing ban on abortion referrals and abortion counseling for Title X recipients	↓ Availability/ Accommodation
May 2018	Domestic gag rule challenged and blocked from implementation pending litigation	↑ Availability/ Accommodation
Jun 2018	VA Mission Act of 2018 provides continuing education for community providers who serve veterans	↑ Appropriateness
Jun 2018	Department of Labor expands reach of Association Health Plans	↓ Affordability
Jul 2018	Circuit court blocks Interim Rules extending religious exemption from ACA contraceptive mandate	↑ Affordability
Aug 2018	HHS shortens Title X funding period from 3 years to 7 months	↓ Availability/ Accommodation
Aug 2018	Centers for Medicare and Medicaid Services extends short-term health plan duration	↓ Affordability
Aug 2018	District Court dismisses Planned Parenthood case against HHS regarding Teenage Pregnancy Prevention Program shift ^†^	↓ Appropriateness
Oct 2018	Brett Kavanaugh confirmed as SCOTUS justice	–
Oct/Nov 2018	Final Rules extending religious exemption from ACA contraceptive mandate released	↓ Affordability
Mar 2019	Domestic gag rule finalized and immediately challenged	↓ Availability/ Accommodation
Mar 2019	Federal judge invalidates Association Health Plans expansion on the grounds that it violates federal tax law ^†^	↑ Affordability
May 2019	Comprehensive Contraceptive Counseling and Access to the Full Range of Methods of Contraception establishes procedures for comprehensive contraceptive counseling for Military Health System beneficiaries	↑ Appropriateness
Jun 2019	Nationwide injunction issued preventing ACA contraceptive mandate enforcement against those with religious objection	↓ Affordability
Jun/Jul 2019	Ninth Circuit Court of Appeals denies stay that would block domestic gag rule, allowing it to begin going into effect	↓ Availability/ Accommodation
Jul 2019	Nationwide injunction issued against ACA contraceptive mandate final rule exemptions	↑ Affordability
Jul 2019	District Court rules to allow extension of short-term limited duration insurance	↓ Affordability
Aug 2019	Over 1,000 clinics, including Planned Parenthood, withdraw from Title X	↑ Availability/ Accommodation
Oct 2019	Petitions filed for Little Sisters of the Poor v. Pennsylvania and Donald J. Trump v. Pennsylvania (both are challenging rulings that block Trump’s religious exemption rules for the ACA contraceptive mandate) ^†^	↓ Affordability

^†^Policy change is ongoing or has been updated after 2019 (see text for more information)

^‡^Impact of policy change according to Levesque et al. [33] dimensions of healthcare access, where ↑ indicates a theoretical increase and ↓ indicates a theoretical decrease in contraceptive access

### Approachability

Policy can impact contraceptive approachability by changing the way that community members perceive the need for contraceptive care and understand how and where such care can be accessed. The 2009 Health Information Technology for Economic and Clinical Health Act (HITECH), enacted as part of the American Recovery and Reinvestment Act, made changes to the administration of the Health Insurance Portability and Accountability Act (HIPAA) of 1996 [35]. This change increased the privacy and security of health data and increased transparency when breaches occur [35]. Later, the Final Omnibus Rule of 2013 filled remaining security and privacy gaps in HIPAA and HITECH regulations [36]. These policy changes relate to contraceptive approachability because they impact the transparency and security of contraceptive care, potentially improving contraceptive approachability by growing trust in health systems and helping community members perceive contraceptive services as beneficial.

Additionally, the Title V Abstinence Education Grant Program, which had supported abstinence-only sex education since 1996, briefly expired in June 2009 [37]. However, it was resurrected in 2010 through the inclusion of Title V funding in the Affordable Care Act (ACA) [37]. It once again expired briefly in September 2017 and was reintroduced and renamed the “Sexual Risk Avoidance Education” program in the Bipartisan Budget Act of 2018 [37, 38]. Another stream of funding for abstinence-only sex education, the Community-Based Abstinence Education Grant Program, which was established in 2000, was eliminated through the Consolidated Appropriations Act of 2011 [37]. This program was later revived as the “Competitive Abstinence Education” program through the Consolidated Appropriations Act of 2012 and renamed the “Sexual Risk Avoidance Education” program in the Consolidated Appropriations Act of 2016 [37, 38]. These policy changes influence contraceptive approachability because they impact the contraceptive education and information available to community members which can change perceptions of the need for and usefulness of contraceptive care.

### Acceptability

Policy changes can impact contraceptive acceptability by influencing the ways that people think and feel about seeking care. For example, changes to structural responses to sexual violence impact contraceptive acceptability by influencing survivors’ ability and willingness to seek care after victimization. In April 2011, the US Department of Education’s Office of Civil Rights sent a document known as the “Dear Colleague letter” to advise colleges and universities of new Title IX of the Education Amendments (Title IX) guidelines, requiring schools to take actions to eliminate campus student harassment and sexual violence [39]. Later, under the Trump administration, US Secretary of Education Betsy Devos eliminated this guidance, requiring a higher evidence standard in sexual assault cases [40]. Meanwhile, one of the original compliance policies established to regulate college handling of sexual assault and harassment, the Clery Act, was amended when the Campus Sexual Violence Elimination Act (Campus SaVE Act) was integrated into the Violence Against Women Reauthorization of 2013, increasing requirements for colleges for sexual assault response and improving survivors’ rights [41, 42]. These policy changes have changed expectations of the ways that colleges prevent and respond to sexual violence. As a result, students may feel differently about reporting sexual violence to university employees. Likewise, university employees have increased guidance in how to respond to reports of sexual violence and refer students to relevant services. Since students experiencing sexual violence often have an increased need for reproductive health services and contraceptive care, these changes are relevant to contraceptive acceptability because they could impact students’ ability and willingness to seek healthcare, including contraception, following victimization.

Additionally, in September 2017, the US Department of Health and Human Services (HHS) announced a draft of the Strategic Plan for 2018 to 2022 [43]. This plan imposed religiously based ideological views, mentioning multiple times that life begins at conception [43, 44]. Despite widespread concerns with this plan, which is meant to guide federal policy over this four-year period, the final version of the Strategic Plan remained largely unchanged and retained references to life beginning at conception [45]. This policy change demonstrates the ways that social norms and religious beliefs can influence policy decisions and ultimately contraceptive access. Furthermore, indoctrinating these values into federal legislation could impact how people view the norms and stigma around the contraceptive care that they seek.

### Availability and accommodation

By influencing the location and density of health facilities and their ability to provide adequate appointments and services, policy also impacts people’s ability to physically reach contraceptive care in a timely manner. For example, in April 2009, the Food and Drug Administration (FDA) lowered the age at which people can access over-the-counter emergency contraception to 17 years old [46]. In 2011, the FDA recommended that the age restriction be eliminated entirely; however, HHS overruled the recommendation, likely in an effort to appease conservatives prior to the announcement of ACA contraceptive mandate rules [46, 47]. In 2013, the emergency contraception brand, Plan B One-Step, was approved for over-the-counter sale regardless of age [46]. In April 2016, market protection for Plan B One-Step expired, allowing over-the-counter sale of all emergency contraception regardless of age [46]. These policy changes are relevant to contraceptive availability and accommodation because they impact when, where, and how people can physically access emergency contraceptive care.

The Neighborhood Health Centers program was developed in the 1960s to provide community-based primary care and was subsequently funded through policies such as the Health Care Safety Net Act of 2008 [48]. In 2010, the ACA established the Community Health Center Fund to expand and operate these community-based health centers which serve many low-income Americans [48]. This fund was initially authorized through 2015 and was extended to 2019 through the Medicare and Child Health Insurance Program Reauthorization Act of 2015 and the Bipartisan Budget Act of 2018 [49]. In response to the COVID-19 pandemic, the fund was extended in 2020 through the Coronavirus Aid, Relief, and Economic Security (CARES) Act and in 2021 through the American Rescue Plan [50, 51]. These policy changes are relevant to contraceptive availability and accommodation because they impact community members’ ability to physically access contraceptive care conveniently, in their local communities.

In December 2016, President Obama finalized an amendment to the Title X Family Planning program eligibility requirements, prohibiting exclusion from subawards for reasons other than the ability to provide services [52]. This amendment took effect in January 2017 but was promptly nullified under a Joint Resolution of Disapproval signed by President Trump [52]. Then, in 2018, HHS shortened the Title X funding period from three years to seven months [53]. Also in 2018, the Trump administration announced what they call the “Protect Life Rule” (known by pro-choice advocates as the “domestic gag rule”) which proposed a ban on abortion referrals and abortion counseling for Title X recipients [54, 55]. This rule was initially challenged and blocked from implementation but ultimately the Ninth Circuit Court of Appeals denied a stay in June 2019, allowing the domestic gag rule to go into effect [56]. In response, over 1,000 clinics withdrew from the Title X program in August 2019 rather than be forced to eliminate abortion referrals and counseling [56]. Recently, the Biden administration reversed the domestic gag rule in October 2021, potentially allowing withdrawn clinics to return to the Title X program [57]. Through funding that can make or break healthcare facilities, these policy changes impact contraceptive availability and accommodation by influencing where people can access contraceptive care and how many local options are available to them. When more facilities are forced to close or reduce their hours and services, community members face fewer options, greater wait times, and less convenient care.

There have also been changes in recent years to military guidelines on healthcare and contraception. The Veterans Access, Choice, and Accountability Act of 2014 expanded healthcare options for veterans with specific “burden[s]” to receive healthcare with “choice” contracted providers [58]. Specific branches of the military have also introduced new policies impacting the availability of contraception for service members. In January 2015, the Navy added walk-in contraceptive clinics and increased the availability of long-acting reversible contraception (LARC) during basic training. In contrast, in January 2016, the Marines restricted the availability of LARC at basic training and began promoting injectable contraceptives over other contraceptive methods [59]. These policy changes are relevant to contraceptive availability and accommodation because they impact when, where, and how military members and veterans physically access contraceptive care and which options are easily and conveniently accessible to them.

### Affordability

By influencing the price of health care and people’s access to health insurance and other financial resources, policy changes can also greatly impact contraceptive affordability. Announced in February 2009 and signed into law in March 2010, the ACA is a US healthcare reform law establishing a new healthcare marketplace and requiring insurance coverage of preventive services, protections for pre-existing health conditions, and allowances for expanded dependent coverage [60–62]. Amendments, including the Women’s Health Amendment and the Health Care and Education Reconciliation Act, added additional protections such as Medicaid expansion and inclusion of women’s preventive care as mandated preventive services [61, 63]. The introduction of the ACA and these amendments that added additional protections are relevant to contraceptive affordability because they increased access to health insurance and reduced the cost of contraception and contraceptive care for many Americans.

The Institute of Medicine released recommendations for preventive services for women in July 2011, which were adopted into the ACA preventive care guidelines, adding contraceptive coverage as a required preventive service [62]. Shortly before implementation of the ACA contraceptive mandate began, an exemption for churches was announced in January 2012 and then extended in February 2012 to other religious employers [64]. Then, the Supreme Court ruled in June 2014 in *Burwell v. Hobby Lobby Stores, Inc.* that for-profit companies with a religious objection to birth control are also exempt from the mandate [65, 66]. Under the Trump administration, the religious exemption was further extended to the point that virtually any moral or religious objection can exempt an employer from the mandate [67]. This most recent change was challenged in court, culminating in the July 2020 Supreme Court decision in *Little Sisters of the Poor Saints Peter and Paul Home v. Pennsylvania* which held that the religious and moral exemptions were lawful [65]. These restrictions on the ACA contraceptive coverage mandate are relevant to affordability because they increase the cost of contraception for Americans who work for exempt employers.

There was also resistance to the Medicaid expansion included in the ACA. In June 2012, the Supreme Court ruled in *National Federation of Independent Business v. Sebelius* to uphold most ACA provisions but made Medicaid expansion optional for states [22, 68]. Optional Medicaid expansion went into effect in January 2014 with 28 states and the District of Columbia participating [69]. This also changed the way that states utilize Section 1115 waivers and State Plan Amendments [70] to expand family planning Medicaid. These changes to Medicaid increased contraceptive affordability for some but left others, in states that did not expand Medicaid, with high contraceptive costs. Contraception was also made more affordable for some young Americans through the National Defense Authorization Act of 2011 which created TRICARE Young Adult, extending military member dependent coverage [71].

In June 2018, the Department of Labor expanded the reach of Association Health Plans which could have decreased consumer protections and allowed companies to sidestep benefit requirements [72]; however, this expansion was challenged and ultimately invalidated by a federal judge in March 2019 on the grounds that it violated federal tax law [72]. Also, under the Trump administration, in 2018 the Centers for Medicare and Medicaid Services extended the duration allowed for short-term health plans, which are not required to comply with the ACA [73]. This was also challenged in court, but a District Court ruled in 2019 to allow the extension [73]. This change was relevant to contraceptive affordability because it impacts the cost of contraception and contraceptive care by providing avenues for companies to sidestep ACA contraceptive coverage requirements.

### Appropriateness

Policies and mandates that influence how providers are trained and prepared to meet patients’ needs can impact people’s ability to engage with evidence-based contraceptive care. The Teen Pregnancy Prevention Program (TPPP), providing competitive grant funding for programs that reduce teen pregnancy, was initially established in December 2009 in the Consolidated Appropriations Act of 2010 [74]. Subsequent appropriations laws continued funding this program until TPPP grant projects beginning in 2015 were shortened by two years, ending in 2018 rather than 2020 [74, 75]. In April 2018, a district court granted Planned Parenthood continued participation in the TPPP [76]*.* However, four days before this decision, an HHS Funding Opportunity Announcement shifted the TPPP to promote abstinence-only sex education and decrease focus on evidence-based approaches [74]. When Planned Parenthood sued HHS over this shift, a district court dismissed the case in August 2018 [77]. However, in January 2020, the US Court of Appeals for the Ninth Circuit ruled that these TPPP changes were unlawful because they are counter to the TPPP emphasis on evidence-based programming [78]. When rooted in evidence-based programming, the TPPP improves the appropriateness of contraceptive care by funding many initiatives that prevent unwanted pregnancies and increase understanding of barriers to care, ultimately helping providers and facilities better meet community contraceptive needs.

In the past decade, several guidelines and recommendations, including the US Medical Eligibility Criteria for Contraceptive Use (released June 2010, updated July 2016) [79], the US Selected Practice Recommendations (released June 2013, updated July 2016) [80], and the Providing Quality Family Planning Services recommendations (released April 2014, updated March 2016) [81] were published, increasing the appropriateness of contraceptive care by providing healthcare providers with recommendations on the safe use of contraceptive methods. Similarly, in December 2010, the Healthy People 2020 initiative also increased the appropriateness of care by providing science-based national goals and objectives to guide national health promotion efforts in the United States [82]. Additionally, in 2015, updates to the Indian Health Service guidelines made emergency contraception more accessible to American Indian and Alaska Native community members [83]. These regulations have increased the appropriateness of contraceptive care by better preparing providers and facilities to meet patient and community contraceptive needs.

Several policy changes have also increased contraceptive appropriateness for military members and veterans. The VA Mission Act of 2018 increased the appropriateness of care by providing continuing education for community providers who serve veterans [58]. Similarly, in 2019, the Defense Health Agency [84] established procedures for comprehensive contraceptive counseling with Military Health System beneficiaries through the policy entitled Comprehensive Contraceptive Counseling and Access to the Full Range of Methods of Contraception. Each of these policies improved the appropriateness of contraceptive care through standardized training procedures that better prepare providers to engage in contraceptive counseling with veteran/military populations.

## Discussion

These findings have suggested that over the past decade, federal US policies relevant to contraceptive access have largely followed partisan divides, with contraceptive access increasing in years with a Democratic president (President Obama) and decreasing while a Republican president (President Trump) was in office. This finding is not surprising given the party-specific polarization present in reproductive politics in the US [23–28]. This study showed that there were more policy changes related to the affordability (e.g., ACA contraceptive mandate) and physical availability (e.g., Veterans Access, Choice, and Accountability Act; changes to Title X) of contraceptive care than other aspects of contraceptive access. This is likely, in part, a response to perceptions of these dimensions of access as primary barriers to care (e.g., [6, 7]).

There have also been many policy changes related to recent updates to the ACA and the Title X Family Planning Program, which largely impact the affordability and availability of contraceptive care. In fact, out of the 70 direct-impact policy changes identified in this analysis, 20 (29%) were related to the ACA and 9 (13%) were related to Title X. Both policies have enormous impact on contraceptive access in the United States, and the many changes to them throughout the past decade, which have largely ebbed and flowed with election cycles according to partisan divides, reflect the political polarization present in the American public [85]. This legislative back-and-forth carries real-world implications for contraceptive care, breaking down and reproducing barriers to care and creating confusion for both those seeking and providing contraceptive care. For example, research shows that policy complexity, frequent policy changes, and poor public communication, can lead to public confusion and misinterpretation of the law [86–88]. In the case of the ACA, this confusion can lead to delays in care [89], indicating a need for policy stability and campaigns to increase public understanding of healthcare policy.

## Implications for practice and/or policy

This study identified policy needs and practice gaps that could be addressed to improve contraceptive access across the dimensions of healthcare access. First, eliminating abstinence-only sex education policies and Sexual Risk Avoidance Education programs and instead promoting comprehensive sex education would improve contraceptive access by increasing available contraceptive information and decreasing stigmatizing rhetoric. Additionally, continuing to extend the Community Health Center Fund would increase the availability and affordability of contraceptive care. The 2021 removal of the domestic gag rule may allow clinics including Planned Parenthood to reenter the Title X program, which would also greatly increase the availability, affordability, and appropriateness of care. Future policy action should continue to provide funding for these clinics to deliver comprehensive family planning care.

There is also a need for increased transparency regarding care options and costs of services as well as amplified outreach efforts to combat misinformation and confusion created by continuous changes to key family planning policies such as Title X and the ACA. Such efforts are key avenues for improving contraceptive access by increasing knowledge about healthcare policy and opportunities for care in local communities. Moreover, years of medical mistreatment of Black, Indigenous, and people of color (BIPOC) communities [16, 90] and continued implicit and explicit bias in healthcare [91, 92] has generated an earned distrust in medical systems among these communities (e.g., 93). This distrust represents a critical barrier to contraceptive access, impacting the ways that BIPOC perceive the need for care, seek or avoid care, and engage with healthcare systems [93]. With the possible exception of the 2015 update to the Indian Health Service guideline, which sought to increase emergency contraceptive access for American Indians and Alaska Natives, none of the policies reviewed in this study directly address the issue of discrimination and related distrust and decreased access in contraceptive care. As such, there is an immense and immediate need for policy and practice action that addresses both the interpersonal and structural presence of discrimination in healthcare and begins to inspire trust by developing culturally responsive community outreach and contraceptive care.

Finally, considering the Supreme Court decision upholding extensive moral and religious objections to the ACA contraceptive mandate, a focus on increasing options for those affected employees could increase contraceptive access. Creative solutions in both policy and practice arenas are needed to ensure contraceptive access for all Americans, including those with employers who are exempted from the ACA contraceptive mandate.

## Limitations and future directions

One limitation of the current study is that it is possible and even likely that these methods did not identify all relevant federal policies that have recently influenced contraceptive access. The grey literature review was meant to capture the most salient policy changes related to recent contraceptive access, so any policy changes not described here may not be as relevant as those included in the current summary but could be investigated in future studies. Additionally, the current study was limited to examining contraceptive access, but many other recent policy changes have shifted the family planning policy landscape. It is often difficult to compartmentalize aspects of family planning care because true healthcare access requires the availability of comprehensive family planning services, including contraceptive, abortion, fertility, and pregnancy services [94]. Exploring the comprehensiveness of care rather than considering these services in silos can change the ways that policies are analyzed. For example, the Title X domestic gag rule and the resulting clinic withdrawal from the Title X program [56] were coded in this analysis as “availability and accommodation” because they greatly impacted the physical availability of contraceptive care by reducing the funds available to providers and decreasing clinics’ ability to provide widespread contraceptive services. However, this policy change is also related to the appropriateness of overall family planning care. When clinics withdrew from the Title X program rather than eliminate abortion counseling and referrals, they were upholding family planning access by providing appropriately comprehensive services. Thus, future research could explore the role of recent policies on abortion access and the overall comprehensiveness of family planning care. Furthermore, the example of changes to Title X impacting both contraceptive availability/accommodation and appropriateness illustrates the overlap between the healthcare access dimensions, which can make it challenging to categorize policies into only one dimension. The current study sought to organize and analyze the identified policy changes according to the most salient healthcare access dimension. Future research could extend this analysis by narrowing the focus to a single policy and exploring its impact on multiple healthcare access dimensions. Additionally, whereas the current study focused on US federal policy, future research could investigate the role of state-level policies on contraceptive access and family planning access more generally. Finally, this study utilized grey literature and considered the theoretical impact of recent policy changes on contraceptive access. Future research could expand on these findings by reviewing, summarizing, and building on empirical research on this topic.

## Conclusion

This study has provided an overview of recent US policy changes related to contraceptive access and analyzed their theoretical impact on contraceptive access. Using Levesque et al.'s (2013) five dimensions of healthcare access [33], findings indicated that most policy changes impacted the affordability, physical availability, and appropriateness of contraceptive care and that recent policy changes have largely fluctuated with election cycles according to partisan divides. In particular, many policy changes were related to the ACA and Title X. The implementation of the ACA and subsequent updates to it have increased the affordability of contraception, whereas changes to Title X have decreased the availability and appropriateness of contraceptive care. Opportunities to ensure contraceptive access for all Americans include promoting comprehensive sex education, extending the Community Health Center Fund, increasing contraceptive care options for people with employers who are exempted from the ACA contraceptive mandate, addressing discrimination and building trust in contraceptive care, and amplifying outreach efforts to combat misinformation and confusion created by continuous changes to key family planning policies. Continued research on the role of policy in determining reproductive autonomy is warranted, and practice and policy action is needed to improve contraceptive access.

## Data Availability

The grey literature sources used to create the policy timeline for this study are available from the corresponding author on reasonable request.

## Abbreviations

ACA: Affordable Care Act
Title X: Title X of the Public Health Services Act
HITECH: Health Information Technology for Economic and Clinical Health Act
HIPAA: Health Insurance Portability and Accountability Act
Title IX: Title IX of the Education Amendments
HHS: US Department of Health and Human Services
FDA: Food and Drug Administration
LARC: Long-active reversible contraception
TPPP: Teen Pregnancy Prevention Program
BIPOC: Black, Indigenous, and people of color
